# A comprehensively characterized cell line panel highly representative of clinical ovarian high-grade serous carcinomas

**DOI:** 10.18632/oncotarget.9929

**Published:** 2016-06-10

**Authors:** Kelsie L. Thu, Mahboubeh Papari-Zareei, Victor Stastny, Kai Song, Michael Peyton, Victor D. Martinez, Yu-An Zhang, Isabel B. Castro, Marileila Varella-Garcia, Hanquan Liang, Chao Xing, Ralf Kittler, Sara Milchgrub, Diego H. Castrillon, Heather L. Davidson, C Patrick Reynolds, Wan L. Lam, Jayanthi Lea, Adi F. Gazdar

**Affiliations:** ^1^ British Columbia Cancer Agency Research Centre and University of British Columbia, Vancouver, BC, Canada; ^2^ Hamon Center for Therapeutic Oncology Research, UT Southwestern Medical Center, Dallas, TX, USA; ^3^ School of Chemical Engineering and Technology, Tianjin University, Tianjin, P.R. China; ^4^ Division of Medical Oncology, University of Colorado Denver School of Medicine, Aurora, CO, USA; ^5^ Eugene McDermott Center for Human Growth & Development, UT Southwestern Medical Center, Dallas, TX, USA; ^6^ Simmons Comprehensive Cancer Center, UT Southwestern Medical Center, Dallas, TX, USA; ^7^ Department of Pathology, UT Southwestern Medical Center, Dallas, TX, USA; ^8^ Department of Pathology and Simmons Comprehensive Cancer Center, UT Southwestern Medical Center, Dallas, TX, USA; ^9^ Cell Biology & Biochemistry, Internal Medicine, and Pediatrics, School of Medicine Texas Tech University Health Sciences Center, Lubbock, TX, USA; ^10^ Obstetrics & Gynecology, UT Southwestern Medical Center, Dallas, TX, USA; ^11^ Hamon Center for Therapeutic Oncology Research, Department of Pathology and Simmons Comprehensive Cancer Center, UT Southwestern Medical Center, Dallas, TX, USA

**Keywords:** high-grade serous ovarian carcinoma, cell models, patient-derived xenograft, exome-sequencing, genomic characterization

## Abstract

Recent literature suggests that most widely used ovarian cancer (OVCA) cell models do not recapitulate the molecular features of clinical tumors. To address this limitation, we generated 18 cell lines and 3 corresponding patient-derived xenografts predominantly from high-grade serous carcinoma (HGSOC) peritoneal effusions. Comprehensive genomic characterization and comparison of each model to its parental tumor demonstrated a high degree of molecular similarity. Our characterization included whole exome-sequencing and copy number profiling for cell lines, xenografts, and matched non-malignant tissues, and DNA methylation, gene expression, and spectral karyotyping for a subset of specimens. Compared to the Cancer Genome Atlas (TCGA), our models more closely resembled HGSOC than any other tumor type, justifying their validity as OVCA models. Our meticulously characterized models provide a crucial resource for the OVCA research community that will advance translational findings and ultimately lead to clinical applications.

## INTRODUCTION

Ovarian cancer (OVCA) ranks as the fifth deadliest cancer affecting women in the United States [1]. The dismal prognosis is attributable to a lack of early detection methods, effective treatment strategies, and the current inability to overcome acquired drug resistance [2]. High-grade serous ovarian carcinoma (HGSOC) is the most common and aggressive OVCA subtype [3]. Although comprehensive genomic characterization of HGSOC tumors has provided a catalogue of somatic alterations, their biological relevance and therapeutic potential remain to be thoroughly investigated [4]. An understanding of how these and prospectively discovered genetic alterations contribute to ovarian tumorigenesis is imperative for the development of new therapies targeting tumor biology [2].

To evaluate the biological significance of molecular alterations in OVCA tumorigenesis, clinically and genetically characterized cell models that recapitulate clinical OVCA are required [5, 6]. Without representative cell models, *in vitro* derived findings have limited potential to translate to *in vivo* systems in the early stages of preclinical OVCA research [6–8]. Recently, Domcke *et al*. compared genomic profiles of clinical HGSOC tumors with commonly used HGSOC cell lines and revealed striking dissimilarity [8]. Only 12 infrequently used cell lines were classified as suitable HGSOC models [8], which is a relatively small number compared to other cancer types [9–11]. Moreover, this number is too small to capture the broad spectrum of heterogeneity observed in HGSOC [4, 5, 7, 12, 13]. This study and other recent reports have raised awareness that additional comprehensively characterized, readily available, and representative HGSOC models are needed to facilitate translational OVCA research [6, 8, 14–17].

To address this need, we sought to generate a new panel of genomically characterized OVCA cell lines that accurately model clinical tumors. Unlike another recent group [18], we focused on generating HGSOC cell lines from malignant ascites, a common form and presentation of OVCA [19, 20], using standard media and growth conditions. We created 18 cell lines and 3 patient derived xenografts that display a high concordance in copy number, methylation, gene expression, and mutational profiles with the tumors they were derived from. These models are also highly similar to HGSOC tumors from the Cancer Genome Atlas (TCGA) cohort. Most importantly, we offer these comprehensively characterized, early passage cell lines and xenografts to the research community in hopes of providing a new resource to facilitate advances in OVCA research that will ultimately lead to improved patient outcomes.

## RESULTS

### Establishment of comprehensively characterized OVCA cell lines

**Figure 1:**
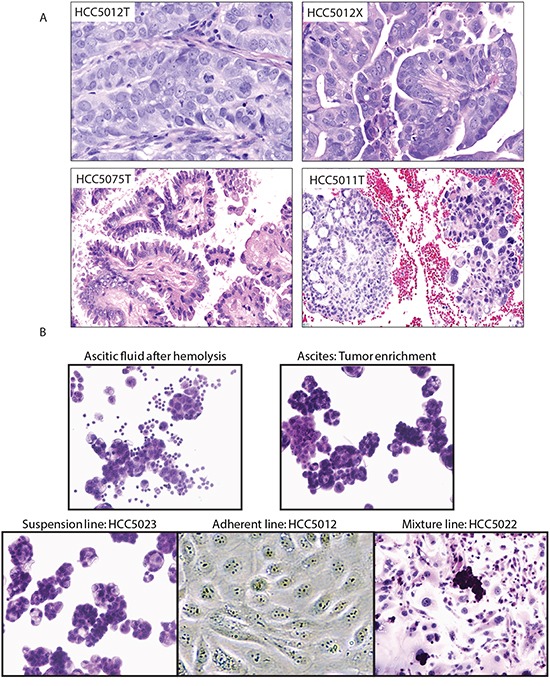
Representative histologies and cell lines generated from OVCA malignant effusions **A.** Histological appearances of tumors. High-grade serous ovarian carcinoma HCC5012 tumor (upper left) and corresponding xenograft (upper right). The appearance of the xenograft is identical to the original tumor. Low-grade serous ovarian carcinoma HCC5075 (lower left). HCC5011 (right lower), high-grade serous ovarian carcinoma (right part of figure) arising from a low-grade serous ovarian carcinoma (left part of figure). This is a rare but well documented occurrence [41]. **B.** Tumor enrichment and established cell lines. Cell lines were generated from tumor cell-containing ascites obtained from malignant effusions. Images of cell preparations at various stages of cell line generation are shown.

We generated and characterized 18 cell lines, 15 patient-matched lymphoblastoid lines, and 3 patient-derived xenografts from 18 treatment naïve OVCA tumor ascites, providing a new panel of suitable models for preclinical research. We achieved a success rate of 41% (18/44 malignant ascites were established as cell lines) with standard media and culture conditions. Of these 18 lines, 6 (33%) were capable of growth as tumor xenografts in mice: HCC5006, HCC5012, HCC5023, HCC5044, HCC5048, and HCC5076 (HCC5012X, HCC5023X, and HCC5048X were genomically characterized, Table 1). Our method enabled us to obtain, enrich and cryopreserve large numbers of original tumor cells for comparison with the corresponding cell lines. Clinical information and culture characteristics for each model are summarized in Table 1 and Supplementary Table 1. Figure 1 illustrates a malignant effusion sample after hemolysis, ascites tumor enrichment, and representative cell lines generated. For each line we have determined a short tandem repeat (STR) profile to enable cell line identification (Supplementary Table 2). Expression levels of *EPCAM* and the characteristic OVCA markers *MUC16*, *WT1*, and *PAX8* were high in tumors, cell lines and xenografts, demonstrating that our models maintain an epithelial phenotype and typical OVCA marker expression (Supplementary Figure 1) [21–24]. Furthermore, serum CA125 was detected at high levels in all patients except for HCC5030, and mRNA levels of its gene (*MUC16*) in corresponding models were also high, suggesting plasma CA125 in xenograft bearing mice could be used as an indicator of therapeutic efficacy [23, 24] (Supplementary Table 3). We proceeded with comprehensive genomic characterization to 1) assess how well cell lines represent corresponding parental tumors, 2) assess similarity to HGSOC tumors in The Cancer Genome Atlas (TCGA) [4], and 3) enable researchers to select lines with the most appropriate genetic backgrounds. All but one line (HCC5075) exhibited a strong resemblance to clinical HGSOC (Table 1, Figure 1).

**Table 1:** Summary of the 51 UTSW specimens

Sample	Age	Stage	Histology	Ethnicity	Key Mutations^≠^	SKY	SNP	Methylation	Expression	Exome-sequencing
HCC5006CL	55	IV	HGSOC	Caucasian	TP53		□			□
HCC5006T					TP53		□			□
HCC5011CL	52	IIIC	HGSOC arising from LGSOC	Caucasian	TP53, BRCA1, MECOM		□			□
HCC5011T					TP53, BRCA1, MECOM		□			□
HCC5012CL	58	IIIC	HGSOC	African American	TP53	□	□	□	□	□
HCC5012T					TP53	□	□	□	□	□
HCC5012X (TX-OV-143X)					TP53	□	□			□
HCC5018CL	57	IIIC	HGSOC	Caucasian	None	□	□	□	□	□
HCC5018T					Not Sequenced	□	□	□		
HCC5019CL	59	IV	HGSOC	African American	TP53, CSMD3	□	□	□	□	□
HCC5019T					TP53, CSMD3	□	□	□	□	□
HCC5020CL	43	IV	HGSOC	African American	TP53, NF1	□	□			□
HCC5022CL	45	IIIC	HGSOC	Hispanic	TP53		□	□	□	□
HCC5022T					TP53		□	□	□	□
HCC5023CL	46	IV	HGSOC	Asian	TP53, RB1	□	□	□	□	□
HCC5023T					TP53, RB1	□	□	□	□	□
HCC5023X (TX-OV-075X)					TP53, RB1, BRCA2, ARID1A	□	□			□
HCC5024CL	60	IIIC	HGSOC	African American	TP53	□	□	□	□	□
HCC5024T					TP53	□	□	□	□	□
HCC5030CL	74	IV	LGSOC (cytology only)^%^	African American	TP53	□	□	□	□	□
HCC5030T					Not Sequenced	□	□	□	□	
^#^HCC5032CL	44	IIIC	^#^HGSC of Mullerian origin	Hispanic	TP53		□	□	□	□
^#^HCC5032T					TP53		□	□		□
HCC5036CL	47	IIIC	HGSOC	Hispanic	TP53	□	□	□	□	□
HCC5036T					TP53	□	□	□	□	□
HCC5044CL	63	IVB	HGSOC	Asian	TP53, PIK3CA		□			□
HCC5048CL	43	IIIB	HGSOC	Caucasian	TP53	□	□	□	□	□
HCC5048T					TP53	□	□	□	□	□
HCC5048X (TX-OV-132X)					TP53, CSMD3, CDK12, MECOM, ARID1A		□			□
HCC5050CL	46	IIIC	HGSOC	Hispanic	TP53, PIK3CA, ARID1A	□	□	□	□	□
HCC5050T					TP53, PIK3CA, ARID1A	□	□	□	□	□
HCC5075CL	45	IIIC	LGSOC	Hispanic	NF1, KRAS		□			□
HCC5075T					NF1, KRAS		□			□
HCC5076CL	41	IV	HGSOC	Hispanic	TP53		□			□
HCC5076T					TP53		□			□
HCC5079CL	61	IIIC	HGSOC	Hispanic	TP53		□			□
	**Total**:	**21**	**36**	**22**	**20**	**34**

^≠^A source of non-malignant cells was sequenced in all cases except HCC5048 to provide a reference for defining somatic mutations

^%^ Low confidence tumor histology due to LGSOC classification based on cytology only, however the molecular profile is consistent with HGSOC

^#^ Subsequent clinical pathology reassigned this case as endometrial cancer, although the molecular profile is consistent with HGSOC

For xenografts (X), the additional sample name (TX-OV-XXXX) indicates the identifier for these samples in the work of Reynolds *et al*., unpublished.

### Cell lines and xenografts maintain the genomic landscape of patient-matched tumors

To determine whether the cell lines and xenografts we established were representative of the tumors they were derived from, we assessed concordance in their genomic profiles. We generated mutation and copy number profiles for every specimen except for two tumors and one lymphoblast line with insufficient material for exome-sequencing, as these data provide the most unique individual signatures (Supplementary Tables 4-6). Clustering and correlation analyses of single nucleotide variant (SNV) data confirmed that cell lines and xenografts more closely resembled patient-matched tumors than unrelated samples (Supplementary Figures 2, 3A-B, two-tailed student's t-test, p<0.0001). The percentage of SNVs detected in tumors that were also detected in matched cell lines/xenografts ranged from 87-97%, indicating the high proportion of tumor variants retained in the cell lines/xenografts generated (Supplementary Figure 4A).

**Figure 2:**
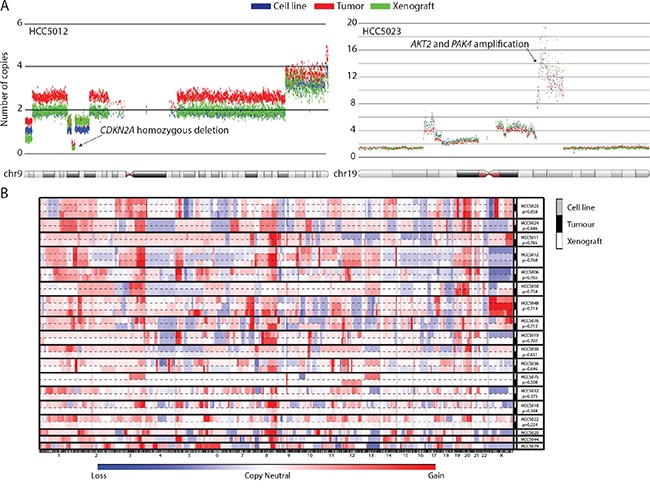
UTSW cell line and xenograft models recapitulate the genomic features of their parental tumors Copy number alteration profiles were unique for each OVCA case, and highly similar between samples derived from the same patient (e.g. tumor, cell line, and xenograft). **A.** Examples of the high level of concordance in copy number alterations detected in primary tumors and their associated models for the HCC5012 and HCC5023 cases. Each dot represents 30 smoothed SNP array probes. Genomic coordinates are plotted on the horizontal axis versus the number of copies for each smoothed data point on the vertical axis. **B.** CNA profiles are plotted against genomic coordinates (horizontal axis) for each UTSW OVCA case. Correlation coefficients representing the similarity between cell lines and the tumors they were derived from are indicated.

Chromosomal instability and copy number alterations (CNAs) are hallmark features of HGSOC [14, 25], and besides mutational profiles, copy number profiles were anticipated to provide the most unique signature for each tumor case. Genome-wide CNA profiles revealed 11/15 tumor-cell line pairs showed very strong concordance, with correlation coefficients, ρ >0.6 (Pearson test, p<0.0001, Figure 2B, Supplementary Figures 3C, 4B and 4C). The 3 tumor xenografts (HCC5012, HCC5023, and HCC5048) we profiled also showed good concordance with their corresponding tumors (Pearson correlation, ρ >0.5, Figure 2B, Supplementary Figures 3-4). Of the tumor-cell line pairs with lower correlation coefficients, 4 had few copy number and loss of heterozygosity (LOH) alterations detected in the tumors; this likely indicates low tumor relative to non-malignant cell content (HCC5018, HCC5022, HCC5030, and HCC5032) (Figure 2B, Supplementary Figure 4B-4D, Supplementary Table 7). We excluded these tumors from further tumor-cell line comparisons for this reason. Cell line and xenograft copy number profiles were also more correlated between patient-matched tumors than unmatched samples, as were DNA methylation and gene expression profiles generated for a subset of cases (Supplementary Figure 3C-3E; two-tailed student's t-test, p<0.0001). Spectral karyotyping of 9 cases also revealed similarity in complex chromosomal rearrangements and tumor cell ploidy (Supplementary Figure 5, Supplementary Table 8). Collectively, our multi-dimensional genome-wide profiling supports the conclusion that the cell lines and xenografts we generated are highly representative of the tumors they were derived from. The high genomic instability, low mutational burden, specific mutations and expression of typical OVCA markers observed in our cell lines were highly consistent with HGSOC, with the exception of one case [14, 21–25]. HCC5075 had pathological and molecular features consistent with a low-grade serous ovarian cancer (Table 1) [14, 25]. Additionally, one case (HCC5032) was confirmed to be of endometrial origin upon detailed histopathological review after manuscript submission (Table 1).

### Established cell lines and xenografts faithfully model HGSOC tumors

**Figure 3:**
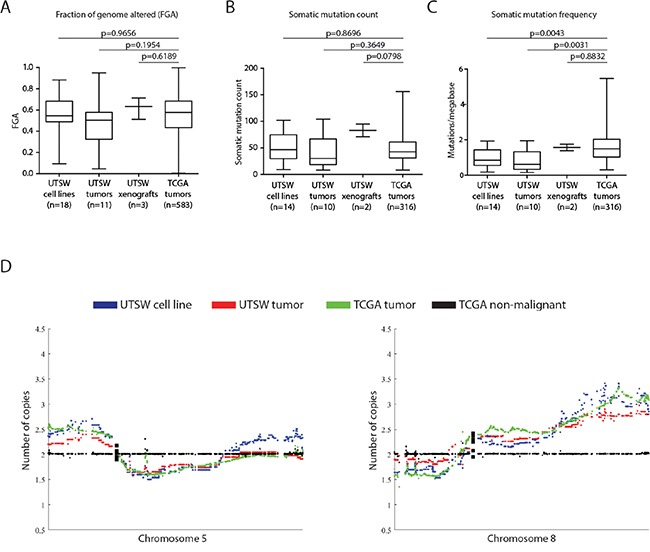
UTSW cell lines highly resemble the TCGA HGSOC cohort across multiple genomic dimensions **A.** The fraction of genome altered (FGA) was compared between the 18 UTSW cell lines, 11 UTSW tumors, 3 UTSW xenografts, and 583 TCGA HGSOC tumors. No significant differences in the extent of FGA between UTSW samples and TCGA tumors were found (Student's t-test, p>0.05). **B.**, **C.** Comparison of somatic mutation counts and frequencies in UTSW samples versus TCGA tumors. Only functional somatic mutations (i.e. those predicted to have a biological effect on protein function) were considered. We observed no significant differences in the number of somatic mutations detected between UTSW samples and the TCGA tumors (B, Student's t-test, p>0.05). The UTSW cell lines and tumors had slightly lower mutational frequencies (ie. mutations per megabase) of DNA compared to the TCGA tumors (C, Student's t-test, p<0.05), however, given the large number of samples profiled in the TCGA cohort (n=316), the variability in mutational load of TCGA tumors is much larger than the UTSW samples. **D.** Comparison of the mean copy number profiles for UTSW cell lines and tumors with the TCGA HGSOC tumors for chromosomes 5 and 8. Tissue-matched TCGA non-malignant profiles are plotted as a copy-neutral (e.g. diploid) reference (black). UTSW cell line copy number patterns (blue) resemble the UTSW tumors (red), and are highly concordant with those of the TCGA tumors (green). Each plotted dot represents the copy number for an individual gene. Supplementary Figure 7 illustrates copy number patterns for UTSW samples and TCGA HGSOCs for all autosomal chromosomes.

We next investigated how well our models represented the TCGA's HGSOC cohort. Mutational counts and the fraction of genome altered by CNAs (FGA) in cell lines, xenografts, and tumors were comparable to those of TCGA tumors (Figure 3A–3B, two-tailed student's t-test, p>0.05). Our cell lines and tumors had lower mutational loads (i.e. mutations/megabase) than TCGA tumors (Figure 3C, Student's t-test, p = 0.004 and p=0.003, respectively) although the range observed in TCGA tumors was large. Despite OVCA tumors having few recurrent mutations besides *TP53* [4], we observed HGSOC-characteristic mutations among the cell lines and xenografts we derived (Table 1, Supplementary Tables 4-5). *TP53* mutations were identified in 16/18 cases, and *BRCA1*, *BRCA2*, *CSMD3*, *CDK12*, *NF1*, *RB1*, and *MECOM* mutations were also detected among the models, illustrating the spectrum of genetic heterogeneity we captured. Furthermore, we observed significant positive correlations between our cell lines and xenografts with the mean copy number (ρ range: 0.12-0.62), methylation (ρ range: 0.39-0.70), and gene expression profiles (ρ range: 0.43-0.61) of TCGA OVCA tumors (Pearson test, p<0.0001, Supplementary Figure 6). Notably, the magnitudes of the positive correlation coefficients we observed across the various cell lines/xenografts and data dimensions were similar to those observed for the CCLE lines classified as good models of HGSOC by Domcke *et al*. [8]. We also observed an impressive concordance in global CNA patterns between our models and the TCGA tumors (Figure 3D, Supplementary Figure 7). GISTIC-based analysis of high magnitude CNAs [26] identified alterations typical of clinical OVCA tumors, including *MYC*/*AKT1* amplifications and *CDKN2A*/*PTEN* deletions (Supplementary Figure 8, Supplementary Tables 9-11) [4]. Our models exhibit low mutation loads and high levels of genomic instability which indicates that their genomic landscapes are typical of HGSOC tumors [8, 14, 27, 28].

Furthermore, we characterized the response of 8 cell lines to standard chemotherapeutics used to treat OVCA, cisplatin and paclitaxel. Adherent cell lines were assessed because they generate reproducible results in dose-response assays. The range in cell line sensitivities was consistent with those reported in the Supplementary Material of Ince *et al*. [18] (Supplementary Table 11). There was no correlation observed between *in vitro* cell line response to cisplatin or paclitaxel and clinical patient response (data not shown). However, this is not surprising given that cell lines are excellent vehicles for predicting response to relevant targeted therapies, but less predictive for response to non-targeted chemotherapeutic agents, as has been described before [5].

### UTSW ovarian cell lines are more similar to OVCA than several other TCGA tumor types

**Figure 4:**
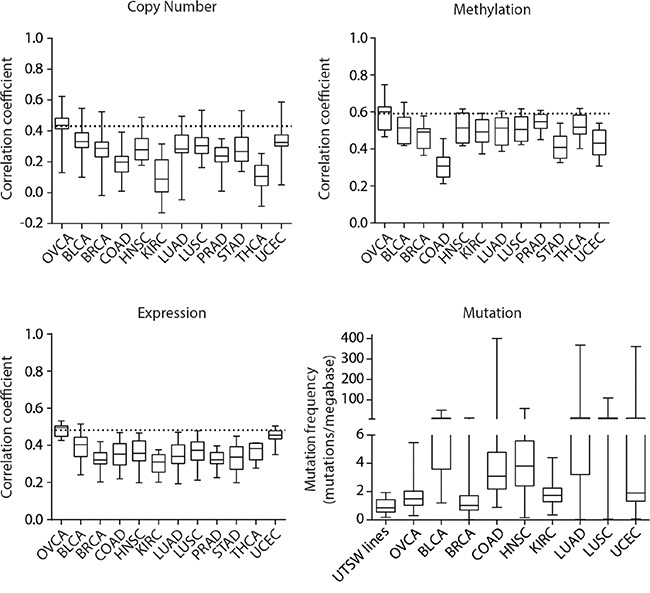
Pan-cancer genomic comparisons of UTSW OVCA cell lines and TCGA tumor types Correlation analyses to assess the similarity between UTSW cell lines and the mean genomic profiles of various TCGA tumor types were conducted for copy number, DNA methylation, gene expression, and mutation data. The 2000 most variably methylated or expressed genes were assessed. For copy number, methylation, and expression plots, the horizontal dotted line indicates the average correlation coefficient observed between the TCGA OVCA tumors and UTSW cell line comparison. For mutation data, functional, somatic mutation frequencies were compared. The TCGA tumor types are indicated as follows: OVCA, bladder urothelial carcinoma (BLCA), breast invasive carcinoma (BRCA), colon adenocarcinoma (COAD), head and neck squamous cell carcinoma (HNSC), kidney renal clear cell carcinoma (KIRC), lung adenocarcinoma (LUAD), lung squamous cell carcinoma (LUSC), prostate adenocarcinoma (PRAD), stomach adenocarcinoma (STAD), thyroid carcinoma (THCA), and uterine corpus endometrial carcinoma (UCEC).

Given the known issues of cross-contamination and poor histological annotation of cell lines from different tissue origins [5, 6, 16, 29], we next aimed to determine whether the lines we created were more strongly correlated to OVCA tumors than to other TCGA tumor types. We again investigated the correlations between gene copy number, methylation and expression profiles of our cell lines with the mean profiles of various TCGA tumor types. We also compared the mutation counts observed in our cell lines to other TCGA tumor types by considering somatic mutations predicted to have a functional impact. These analyses demonstrated that our cell lines most closely resemble OVCA compared to several other epithelial tumor types in each genomic dimension we assessed (Figure 4).

## DISCUSSION

Cancer cell lines and patient-derived xenografts are invaluable tools for studying tumor biology and are widely used to test new anticancer drugs in a preclinical setting [5, 9, 10, 17, 29–31]. However, it is now appreciated that for cell lines and xenografts to be clinically relevant and reliable cancer models, they must be well characterized and recapitulate the genomic landscapes typical of clinical tumors [5–8, 29, 30]. A number of reports have articulated the need for new OVCA models to improve translational research, raising numerous concerns with currently available models including: misidentification and/or contamination, lack of resemblance to clinical tumors, lack of molecular characterization, and lack of availability of the most suitable models [6–8, 15–18, 32, 33]. A revealing study by Domcke *et al*. found that only 12 of 47 OVCA cell lines are suitable HGSOC models, and that they are used in only 1% of studies reporting on HGSOC [8]. In their report, the authors proposed that newly generated, well-characterized HGSOC cell lines from treatment naïve patients could greatly benefit the OVCA research community by improving the value of preclinical discoveries [8].

To address this need, we generated a new panel of comprehensively characterized models which are highly representative of clinical HGSOC tumors with the exception of one case (HCC5075 was *TP53* wild-type, *KRAS* mutant, and had low genomic instability which are features are consistent with low-grade serous ovarian carcinoma [14, 25, 27, 34, 35].). Further histopathological characterization after manuscript submission also revealed that one case (HCC5032) was an endometrial tumor, although its genomic landscape was strongly correlated to those of HGSOC. We have provided multi-faceted genomic evidence to demonstrate that the models we have generated genuinely resemble their parental primary tumors and clinical HGSOCs. Improving on a recently described panel of OVCA cell lines and xenografts [18], the models we have generated: i) include corresponding non-malignant lymphoblastoid cell lines (as sources of constitutional DNA), ii) can be cultured with simple media and growth conditions that are economical and highly amenable for high-throughput drug screening, and iii) include additional dimensions of genomic characterization (namely, whole-exome sequencing, genome-wide DNA methylation, and SKY profiling). Importantly, we have cryopreserved numerous vials of each cell line at low passages and we have deposited the genomic data to share with the research community (GEO Accession ID: GSE71525, SRA BioProject ID: PRJNA291290). The patient-derived xenografts we have generated will also be available for use as *in vivo* models of HGSOC. Finally, we have also cryopreserved multiple vials of the original tumor cell pellets to enable experiments using the primary tumor cells in addition to the corresponding cell lines or xenografts.

Our study sets an unprecedented standard for generating comprehensively characterized cancer cell lines to meet current research needs. In the era of precision medicine [36], it is essential to select representative experimental models that recapitulate the molecular features of the tumors being studied. Our preclinical models will be useful not only for studying HGSOC tumor biology, but also for assessing the efficacy of anticancer agents because of the ease with which they can be cultured and their known mutational profiles. Our new HGSOC panel is a much-needed invaluable tool that will enhance the translation potential of *in vitro* findings [15, 29], ultimately leading to improved therapies and outcomes for OVCA patients.

## MATERIALS AND METHODS

Detailed methods describing OVCA model establishment and genomic profiling and analyses can be found in the Supplementary Material. Cell lines and patient-derived xenografts were generated from malignant peritoneal effusions of 18 treatment naïve OVCA patients with informed patient consent at the University of Texas Southwestern (UTSW). Subsequent pathological review revealed that one tumour (HCC5032) was of endometrial origin after manuscript submission (Table 1). Patient matched lymphoblastoid lines for 15 cases were generated from peripheral blood mononuclear cells, cultured ascites fluids, or mesothelial cells from malignant effusions. Cell line sensitivities to cisplatin and paclitaxel were determined using CellTiter 96^®^ Aqueous One Solution Cell Proliferation Assays (Promega) performed according to the manufacturer's instructions.

Genomic profiling including genome-wide copy number (Affymetrix SNP 6 arrays), gene expression (Illumina HT-12v4 BeadChips), DNA methylation (Illumina HM450K arrays) and exome-sequencing (SureSelect Target Enrichment System for Illumina Paired-End Sequencing) was conducted on genomic DNA or RNA extracted from tumors, cell lines, xenografts, or constitutional DNA from lymphoblastoid lines. All genomic data are available at the Gene Expression Omnibus (GSE71525) and Sequence Read Archive (PRJNA291290). Spectral karyotyping (SKY) was performed on a subset of samples as previously described [37]. Genomic data for the TCGA OVCA cohort was obtained from the TCGA Data Portal, the TCGA OVCA publication page (https://tcga-data.nci.nih.gov/docs/publications/ov_2011/) [4], cBioPortal [38, 39], and pan-cancer TCGA data from the Cancer Genomics Browser (CGB) [40]. Detailed materials and methods, including statistical analyses are provided in the accompanying Supplementary Materials.

## SUPPLEMENTARY MATERIALS METHODS FIGURES AND TABLES

























## References

[1] Siegel RL, Miller KD, Jemal A (2015). Cancer statistics, 2015. CA Cancer J Clin.

[2] Bast RC, Hennessy B, Mills GB (2009). The biology of ovarian cancer: new opportunities for translation. Nature reviews Cancer.

[3] Seidman JD, Horkayne-Szakaly I, Haiba M, Boice CR, Kurman RJ, Ronnett BM (2004). The histologic type and stage distribution of ovarian carcinomas of surface epithelial origin. International journal of gynecological pathology.

[4] Cancer Genome Atlas Research Network (2011). Integrated genomic analyses of ovarian carcinoma. Nature.

[5] Gillet JP, Varma S, Gottesman MM (2013). The clinical relevance of cancer cell lines. Journal of the National Cancer Institute.

[6] Jacob F, Nixdorf S, Hacker NF, Heinzelmann-Schwarz VA (2014). Reliable *in vitro* studies require appropriate ovarian cancer cell lines. Journal of ovarian research.

[7] Konstantinopoulos PA, Matulonis UA (2013). Current status and evolution of preclinical drug development models of epithelial ovarian cancer. Frontiers in oncology.

[8] Domcke S, Sinha R, Levine DA, Sander C, Schultz N (2013). Evaluating cell lines as tumour models by comparison of genomic profiles. Nature communications.

[9] MacLeod RA, Nagel S, Scherr M, Schneider B, Dirks WG, Uphoff CC, Quentmeier H, Drexler HG (2008). Human leukemia and lymphoma cell lines as models and resources. Current medicinal chemistry.

[10] Gazdar AF, Girard L, Lockwood WW, Lam WL, Minna JD (2010). Lung cancer cell lines as tools for biomedical discovery and research. Journal of the National Cancer Institute.

[11] Barretina J, Caponigro G, Stransky N, Venkatesan K, Margolin AA, Kim S, Wilson CJ, Lehar J, Kryukov GV, Sonkin D, Reddy A, Liu M, Murray L (2012). The Cancer Cell Line Encyclopedia enables predictive modelling of anticancer drug sensitivity. Nature.

[12] Cooke SL, Ng CK, Melnyk N, Garcia MJ, Hardcastle T, Temple J, Langdon S, Huntsman D, Brenton JD (2010). Genomic analysis of genetic heterogeneity and evolution in high-grade serous ovarian carcinoma. Oncogene.

[13] Schwarz RF, Ng CK, Cooke SL, Newman S, Temple J, Piskorz AM, Gale D, Sayal K, Murtaza M, Baldwin PJ, Rosenfeld N, Earl HM, Sala E (2015). Spatial and temporal heterogeneity in high-grade serous ovarian cancer.

[14] Berns EM, Bowtell DD (2012). The changing view of high-grade serous ovarian cancer. Cancer research.

[15] Vaughan S, Coward JI, Bast RC, Berchuck A, Berek JS, Brenton JD, Coukos G, Crum CC, Drapkin R, Etemadmoghadam D, Friedlander M, Gabra H, Kaye SB (2011). Rethinking ovarian cancer: recommendations for improving outcomes. Nature reviews Cancer.

[16] Korch C, Spillman MA, Jackson TA, Jacobsen BM, Murphy SK, Lessey BA, Jordan VC, Bradford AP (2012). DNA profiling analysis of endometrial and ovarian cell lines reveals misidentification, redundancy and contamination. Gynecologic oncology.

[17] Scott CL, Becker MA, Haluska P, Samimi G (2013). Patient-derived xenograft models to improve targeted therapy in epithelial ovarian cancer treatment. Frontiers in oncology.

[18] Ince TA, Sousa AD, Jones MA, Harrell JC, Agoston ES, Krohn M, Selfors LM, Liu W, Chen K, Yong M, Buchwald P, Wang B, Hale KS (2015). Characterization of twenty-five ovarian tumour cell lines that phenocopy primary tumours. Nature communications.

[19] Smolle E, Taucher V, Haybaeck J (2014). Malignant ascites in ovarian cancer and the role of targeted therapeutics. Anticancer research.

[20] Kipps E, Tan DS, Kaye SB (2013). Meeting the challenge of ascites in ovarian cancer: new avenues for therapy and research. Nature reviews Cancer.

[21] van der Gun BT, Melchers LJ, Ruiters MH, de Leij LF, McLaughlin PM, Rots MG (2010). EpCAM in carcinogenesis: the good, the bad or the ugly. Carcinogenesis.

[22] Zhao L, Guo M, Sneige N, Gong Y (2012). Value of PAX8 and WT1 Immunostaining in Confirming the Ovarian Origin of Metastatic Carcinoma in Serous Effusion Specimens. American journal of clinical pathology.

[23] Bast RC, Badgwell D, Lu Z, Marquez R, Rosen D, Liu J, Baggerly KA, Atkinson EN, Skates S, Zhang Z, Lokshin A, Menon U, Jacobs I, Lu K (2005). New tumor markers: CA125 and beyond. International journal of gynecological cancer.

[24] Karam AK, Karlan BY (2010). Ovarian cancer: the duplicity of CA125 measurement. Nature reviews Clinical oncology.

[25] Bowtell DD (2010). The genesis and evolution of high-grade serous ovarian cancer. Nature reviews Cancer.

[26] Beroukhim R, Getz G, Nghiemphu L, Barretina J, Hsueh T, Linhart D, Vivanco I, Lee JC, Huang JH, Alexander S, Du J, Kau T, Thomas RK (2007). Assessing the significance of chromosomal aberrations in cancer: methodology and application to glioma.

[27] Jones PM, Drapkin R (2013). Modeling High-Grade Serous Carcinoma: How Converging Insights into Pathogenesis and Genetics are Driving Better Experimental Platforms. Frontiers in oncology.

[28] Kandoth C, McLellan MD, Vandin F, Ye K, Niu B, Lu C, Xie M, Zhang Q, McMichael JF, Wyczalkowski MA, Leiserson MD, Miller CA, Welch JS (2013). Mutational landscape and significance across 12 major cancer types. Nature.

[29] Wilding JL, Bodmer WF (2014). Cancer cell lines for drug discovery and development. Cancer research.

[30] Sharma SV, Haber DA, Settleman J (2010). Cell line-based platforms to evaluate the therapeutic efficacy of candidate anticancer agents. Nature reviews Cancer.

[31] Ricci F, Bizzaro F, Cesca M, Guffanti F, Ganzinelli M, Decio A, Ghilardi C, Perego P, Fruscio R, Buda A, Milani R, Ostano P, Chiorino G (2014). Patient-derived ovarian tumor xenografts recapitulate human clinicopathology and genetic alterations. Cancer research.

[32] Anglesio MS, Wiegand KC, Melnyk N, Chow C, Salamanca C, Prentice LM, Senz J, Yang W, Spillman MA, Cochrane DR, Shumansky K, Shah SP, Kalloger SE, Huntsman DG (2013). Type-specific cell line models for type-specific ovarian cancer research. PloS one.

[33] Beaufort CM, Helmijr JC, Piskorz AM, Hoogstraat M, Ruigrok-Ritstier K, Besselink N, Murtaza M, van Ĳcken WF, Heine AA, Smid M, Koudijs MJ, Brenton JD, Berns EM, Helleman J (2014). Ovarian cancer cell line panel (OCCP): clinical importance of *in vitro* morphological subtypes. PloS one.

[34] Bell DA (2014). Low-grade serous tumors of ovary. International journal of gynecological pathology.

[35] Della Pepa C, Tonini G, Santini D, Losito S, Pisano C, Di Napoli M, Cecere SC, Gargiulo P, Pignata S (2015). Low Grade Serous Ovarian Carcinoma: from the molecular characterization to the best therapeutic strategy. Cancer treatment reviews.

[36] Mendelsohn J (2013). Personalizing oncology: perspectives and prospects. Journal of clinical oncology.

[37] Varella-Garcia M, Chen L, Powell RL, Hirsch FR, Kennedy TC, Keith R, Miller YE, Mitchell JD, Franklin WA (2007). Spectral karyotyping detects chromosome damage in bronchial cells of smokers and patients with cancer. American journal of respiratory and critical care medicine.

[38] Cerami E, Gao J, Dogrusoz U, Gross BE, Sumer SO, Aksoy BA, Jacobsen A, Byrne CJ, Heuer ML, Larsson E, Antipin Y, Reva B, Goldberg AP (2012). The cBio cancer genomics portal: an open platform for exploring multidimensional cancer genomics data. Cancer discovery.

[39] Gao J, Aksoy BA, Dogrusoz U, Dresdner G, Gross B, Sumer SO, Sun Y, Jacobsen A, Sinha R, Larsson E, Cerami E, Sander C, Schultz N (2013). Integrative analysis of complex cancer genomics and clinical profiles using the cBioPortal. Science signaling.

[40] Goldman M, Craft B, Swatloski T, Ellrott K, Cline M, Diekhans M, Ma S, Wilks C, Stuart J, Haussler D, Zhu J (2013). The UCSC Cancer Genomics Browser: update 2013. Nucleic acids research.

[41] Vang R, Shih Ie M, Kurman RJ (2009). Ovarian low-grade and high-grade serous carcinoma: pathogenesis, clinicopathologic and molecular biologic features, and diagnostic problems. Advances in anatomic pathology.

