# Supplementary material for: A comprehensively characterized cell line panel highly representative of clinical ovarian high-grade serous carcinomas
Source: Oncotarget. 2016 Jun 10;8(31):50489–99. doi: 10.18632/oncotarget.9929 (PMC5584155; doi:10.18632/oncotarget.9929)
Supplement: Supplementary file 1 [file oncotarget-08-50489-s001.pdf]

A comprehensively characterized cell line panel highly representative of clinical ovarian high-grade serous carcinomas

SUPPLEMENTARY DATA

Ovarian tumor acquisition and cell line generation

Malignant effusions and peripheral blood samples were collected from treatment naïve OVCA patients with informed patient consent at the University of Texas Southwestern (UTSW). Fibrin clots were prevented or reduced by the addition of 1U of heparin per ml of ascites at the time of specimen collection. For clots present at the time of tumor cell enrichment, agitation of the clotted material in PBS, and RBC lysis buffer or for dense clots, brief digestion with a tissue dissociation enzyme (Roche Liberase TM) was used to release the tumor cell clusters. Tumor cells were enriched by two or more cycles of low speed centrifugation plus differential substrate attachment. The percentage of tumor cells in the enriched specimen was estimated by cytology. Initial growth was often limited, and passages occurred at irregular, infrequent intervals, whenever tumor cell density permitted. Tumor cell lines were established after enrichment, and were cultured in ACL4 medium supplemented with 5% fetal bovine serum. The less adherent occasionally clustered tumor cells were further enriched during culture using differential adherence to separate them from the single mesothelial cells.

We defined established cell lines as those capable of continuous growth for at least 6 months in culture and capable of surviving freeze-thaw cycles, with minimal or absent mesothelial cell contamination. The enriched tumor cells were cultured in ACL4 growth media supplemented with 5% fetal bovine serum. Matched non-malignant, immortalized B lymphoblastoid lines were also created for a source of constitutional DNA, by infection of peripheral blood mononuclear cells with Epstein-Barr virus, using a modification of established methodologies [37]. Occasionally, other sources of constitutional DNA included spontaneous B lymphoblastoid lines derived from cultured ascites fluids, or enriched mesothelial cells derived from the malignant effusions. For each patient we generated a primary tumor cell line, and for most patients, we obtained enriched primary tumor cells and a cell line as a source of constitutional DNA. Furthermore, we established patient derived xenografts for 3 patients, by injecting purified tumor cells intraperitoneally into athymic nude mice (performed in Dr. Reynolds' laboratory). Importantly, all cell lines generated have been cryopreserved at multiple early passages and are available to researchers.

The clinical and histopathological features of each patient tumor sample and culturing details regarding established cell lines are indicated in Table 1 and Supplementary Table 1. RNA and DNA extractions from cell pellets were performed using Trizol reagent and the Qiagen DNeasy Blood and Tissue kit, respectively. Only DNA was extracted from the lymphoblastoid lines. Nucleic acid quality was assessed by gel electrophoresis and quantity determined using a NanoDrop spectrophotometer.

Genomic profiling of UTSW ovarian samples

As soon as cell density permitted we prepared large seed stocks for cell line cryopreservation, and cell pellets from these early passage stocks to provide DNA and RNA for molecular profiling. We employed this strategy to limit the genetic drift between primary tumor cells and the derived cell lines.

DNA fingerprinting

For each sample, a DNA fingerprint was determined using a panel of polymorphic short tandem repeats (STRs) to provide a unique signature for each cell line (Supplementary Table 2). The use of STR analysis of specific loci in the human genome has become the standard for cell line identification and contamination detection [10]. The STRs of our cell lines was assessed using the PowerPlex 1.2 System (http://www.promega.com/applications/cellularanalysis/cellauthentication.htm), an inexpensive, easy to use, and reliable kit available from Promega Corporation that combines analysis of nine loci including amelogenin for sex identification of the donor. The ATCC (http://www.atcc.org/en/STR_Database.aspx) and our center maintain databases of the DNA fingerprints of several hundreds of cell lines, providing evidence of their independent origin from human tumors. For the specimens described in this work, we used the tumor fingerprint as the reference fingerprint and confirmed that all cell lines and xenografts were consistent with the STR profile of the tumor (unless no tumor was available in which case the cell line STR was the reference).

Copy number

Genomic DNA from tumors, cell lines, xenografts, and non-malignant lymphoblast lines were hybridized to Affymetrix Genome-Wide SNP6 arrays according to the manufacturer's instructions. Raw SNP data were processed using Partek Genomics Suite Software as previously described [38]. Briefly, probe intensities were corrected for fragment length, GC content, and probe sequence, and were subsequently quantile normalized. Copy number profiles were generated using Partek's Paired Copy Number Analysis workflow using patient matched constitutional DNA as a baseline for defining somatic alterations in each OVCA sample (tumor/cell line/ xenograft). The Genomic Segmentation algorithm was applied with SNR=0.3, a 30 marker minimum segment size, and p-values < 10^{-7} for defining copy number alterations (CNAs). Genomic segments were mapped to NCBI build 37 (hg19) and RefSeq genes.

Spectral karyotyping (SKY)

SKY was performed on metaphase spreads for 10 cases, including 2/9 with tumor/cell line/xenograft trios (HCC5012, HCC5023), 7/9 with tumor/cell line pairs (HCC5018, HCC5019, HCC5024, HCC5030, HCC5036, HCC5048, and HCC5050), and HCC5020, as previously described [39] (Supplementary Table 8). Cells in culture were incubated in Colcemid (0.05 ug/mL) for metaphase arrest for 2h30min, following harvesting, hypotonization in 0.075M KCl for 25 min, and fixation in 3:1 methanol:glacial acetic acid with Cytoclear (5ul/ ml of fixative, ProCell) to remove persistent cytoplasm. Fixed suspensions were dropped onto slides and subjected to the protocol for SKY as described [39]. The human SKY probe cocktail and reagents for hybridization and immunochemical detection were provided by Applied Spectral Imaging (ASI, Vista, CA). Metaphase spreads were imaged using the SD200 SpectraCube system with the SKY-1 optical filter (Chroma Technology, Brattleboro, VT), mounted on an Olympus BX60 microscope (Olympus Corp., Tokyo, Japan). SKYView version 7.2.6 software (ASI) was used for complete analyses of 10 metaphases per specimen. Electronically inverted DAPI images (band images) were also used to identify deletions and intrachromosomal rearrangements, as well as to assign the chromosome breakpoints. Giemsa-Trypsin-Leishman banding was also performed using standard method including incubations at 56°C overnight, 50 sec of trypsin digestion and 4 min of Leishman’s staining. G-banded metaphase spreads were karyotyped using the BandView software version 7.2.6 (ASI). Karyotype interpretation has followed international nomenclature guidelines (ISCN, 2013).

DNA methylation

Genome-wide DNA methylation profiles were generated using the Illumina Human Methylation 450K array, which assays DNA methylation at over 480,000 unique CpG sites throughout the genome. Genomic DNA was bisulphite converted and hybridized to arrays as per the manufacturer's protocol. Raw array data were exported from Illumina's GenomeStudio software and processed using a 450K BeadChip pipeline employing the BioConductor packages 'lumi' and 'methylumi' to generate normalized methylation beta-values [40]. Beta-values represent the percent methylation at the particular CpG site assayed. Methylation profiles were not generated for constitutional DNA obtained from lymphoblast lines because DNA methylation patterns are tissue specific.

Gene expression

Gene expression profiles were generated using Illumina HT-12v4 BeadChips following the manufacturer's instructions. Background corrected raw data was robust spline normalized using BRB-ArrayTools (developed by Dr Richard Simon and the BRB-ArrayTools Development Team) [41] to generate $\log_2$ transformed, normalized expression values. Expression profiles were not generated for the lymphoblast lines given that gene expression patterns are tissue specific.

Exome sequencing

Whole exome sequencing for 48 OVCA samples (Table 1, Supplementary Table 6) was performed by the UTSW McDermott Next Generation Sequencing Core using the SureSelect Target Enrichment System for Illumina Pair-End Sequencing. Data processing and variant calling was performed by the UTSW Medical Center Bioinformatics Core using the BWA alignment tool, and variant calling was performed using the GATK and MuTect pipelines [42, 43]. Functional variants from the GATK pipeline were defined as SNPs predicted to have a functional impact (SNPEFF impact = moderate or high, including non-synonymous coding mutations, variants affecting splice sites, and frameshifts). All variants identified by the GATK pipeline are presented in Supplementary Table 4. Somatic mutations were called in cell lines, xenografts, and tumors using the MuTect pipeline with reference to patient-matched constitutional DNA from lymphoblast lines. High confidence, functional somatic mutations identified by MuTect are presented in Supplementary Table 5, and were defined according to the following criteria: MuTect judgement = KEEP, t_lod_fstar score > 20, and non-synonymous functional impact as predicted by Mutation Assessor [44]. Mutations were

considered present in tumors, cell lines or xenografts if they were detected by either of the variant calling pipelines.

CA125 assessment

CA125 protein and *MUC16* mRNA expression levels were assessed in patient serum (using a standard clinical test) and derived cell lines, respectively. CA125 protein and MUC16 mRNA expression levels were assessed in patient serum and derived cell lines, respectively. The ARCHITECT CA125 II assay, a Chemiluminescent Microparticle Immunoassay (Abbott Laboratories) was used for the quantitative determination of CA125 defined antigen in human serum and plasma. Values > 35 U/mL were considered elevated. For qRT-PCR, total RNA were extracted with the RNA easy plus kit (Qiagen) and cDNA generated using the High-Capacity cDNA Reverse Transcriptase Kit (ABI/Invitrogen). Expression levels were quantified using a *MUC16* Taqman Gene Expression Assay (Hs01065189_m1, 4331182, Invitrogen) and normalized to GAPDH. The relative expression of *MUC16* for a given sample was determined by real time RT-PCR with reference to the average *MUC16* mRNA levels of four different EBV transformed lymphoblast cell lines.

Data submission

Genomic data generated for the UTSW samples has been deposited to the Gene Expression Omnibus (DNA copy number, methylation and gene expression; GEO Accession GSE71525) and Sequence Read Archive (exome sequencing; BioProject ID = PRJNA291290).

Drug testing of ovarian cell lines

Cells were seeded at 1,000 to 2,000 cells per well in 96 well plates. Twenty-four hours later drug was added and cells were incubated for 96 hours. The MTS assay (CellTiter 96® Aqueous One Solution Cell Proliferation Assay from Promega) was performed according to the manufacturer's instructions. All incubations were done in standard tissue culture incubators set at 37°C, 5% CO2, and a humidified atmosphere. Data was imported into DIVISA software and IC_{50}, ED_{50}, and AUC were calculated from the dose response curves. IC_{50} values reported represent the concentration at which the curves reached half the value of the control (no drug); ED_{50} values represent the concentration at which the curves reached half of the total response; and AUC is the area under the curve where the total area is set to 100. If no value was reached for the IC_{50} the highest drug concentration tested was listed. Eight adherent cell lines, as opposed to suspension lines, were chosen for drug response profiling due to their amenability to the viability assay.

The cancer genome atlas (TCGA) genomic profiles

Segmented copy number data (n=583, Affymetrix Genome-Wide SNP 6 array, hg19) for TCGA HGSOC tumors were downloaded from the TCGA Data Portal. Processed DNA methylation data (n=489, Illumina Human Methylation 27K array) for the TCGA ovarian tumor cohort were downloaded from the TCGA ovarian cancer publication page (https://tcga-data.nci.nih.gov/docs/publications/ov_2011/) [4]. Processed gene expression data (n=413, Illumina HiSeq) for the TCGA cohort was obtained from the Cancer Genomics Browser [45]. TCGA tumor mutation counts based on exome-sequencing were downloaded from cBioportal [46, 47], and mutational frequencies (i.e. mutations per megabase) were obtained from data compiled by Kandoth *et al.* [28]. For both mutation count and frequency datasets, mutations were classified as somatic mutations predicted to have a functional impact.

Processed and normalized copy number, HM27 methylation, and Illumina HiSeq expression data for 11 additional tumor types were obtained from the Cancer Genomics Browser (CGB) [45] for pan-cancer genomic analyses. Mutation data for these additional types were also obtained from cBioportal and the Kandoth *et al.* compilation [28, 46, 47]. Mean profiles for the TCGA tumor copy number, DNA methylation and gene expression data were calculated by averaging copy number $\log_2$ ratios, methylation beta-values or gene expression values (RSEM, RNAseq by Expectation-Maximization) across the TCGA tumors, and Pearson correlations with corresponding UTSW data were performed considering all autosomal genes for copy number, and the 2000 most variably methylated or expressed genes in the TCGA HGSOC cohort.

EpCAM, WT1, PAX8, and MUC16 expression analysis

Gene expression of the epithelial marker (*EpCAM*) and typical ovarian cancer markers (*WT1*, *PAX8*, and *MUC16*) were investigated and compared in the UTSW OVCA samples and clinical tumors of lung (LUAD) and ovarian origin from the British Columbia Cancer Agency as part of the Early Detection Research Network (BCCA, GSE32867 [48]) and the TCGA. The EDRN LUAD expression data was generated using a similar Illumina expression platform, enabling comparison of normalized gene expression levels with UTSW samples. First, genes

were compared in UTSW OVCA samples versus BCCA LUAD samples to demonstrate the similarity or difference in expression levels between OVCA and LUAD. These similarities/differences were then assessed in TCGA RNA-sequencing data obtained from the TCGA Data Portal (RNA-seqV2, https://tcga-data.nci.nih.gov/tcga/). Data were further normalized as an expression ratio of the gene of interest relative to the mean of three housekeeping genes (*PSMB2*, *RAB7A*, *VCP* which were selected because they exhibited the most consistent expression levels across non-malignant samples from 429 samples, spanning 15 TCGA tissue types and the UTSW/BCCA samples) [49] and multiplied by a factor of 100 for visualization (Supplementary Figure 1). The further housekeeping gene normalization was done to enable cross-platform and cross-tissue type comparison of expression values from our study and external datasets. Expression levels were statistically compared using a Wilcoxon rank-sum test (U-test), and a $p<0.05$ was considered significant.

Statistical analyses

All statistical analyses were performed in R statistical software and graphs generated using GraphPad Prism v6. All Students' t-tests were two-sided, and significance was defined as $p<0.05$. GISTIC 2.0 was used with default settings to identify significant regions of copy number gain and loss in the 18 OVCA tumor cell lines [26]. Correlations to compare copy number, methylation, gene expression, and binary mutation profiles (i.e. 1 for presence of mutation, 0 for not detected) of UTSW cell lines and their corresponding primary tumors, and UTSW cell lines and TCGA tumors were performed using a Pearson's correlation test using the mean profile of available TCGA tumors, as described by Domcke *et al*. (and above) [8].

REFERENCES

1. Bast RC, Jr., Badgwell D, Lu Z, Marquez R, Rosen D, Liu J, Baggerly KA, Atkinson EN, Skates S, Zhang Z, Lokshin A, Menon U, Jacobs I, Lu K. New tumor markers: CA125 and beyond. International journal of gynecological cancer: official journal of the International Gynecological Cancer Society. 2005; 15:274-281.
2. Karam AK, Karlan BY. Ovarian cancer: the duplicity of CA125 measurement. Nature reviews Clinical oncology. 2010; 7:335-339.
3. van der Gun BT, Melchers LJ, Ruiters MH, de Leij LF, McLaughlin PM, Rots MG. EpCAM in carcinogenesis: the good, the bad or the ugly. Carcinogenesis. 2010; 31:1913-1921.
4. Zhao L, Guo M, Sneige N, Gong Y. Value of PAX8 and WT1 Immunostaining in Confirming the Ovarian Origin of Metastatic Carcinoma in Serous Effusion Specimens. American journal of clinical pathology. 2012; 137:304-309.
5. Eisenberg E, Levanon EY. Human housekeeping genes, revisited. Trends in genetics: TIG. 2013; 29:569-574.

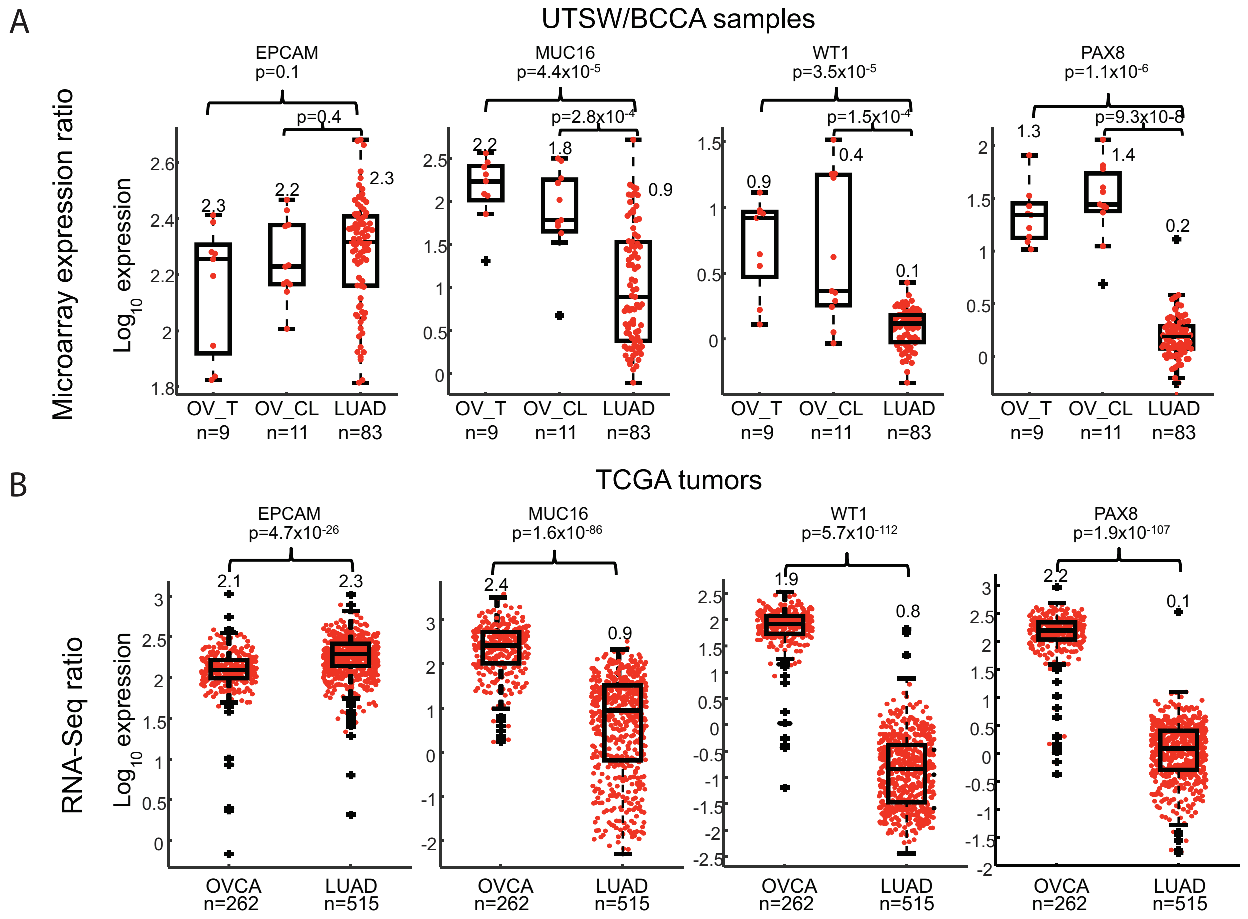

Supplementary Figure S1: UTSW models exhibit expression patterns consistent with epithelial and ovarian origin. *EpCAM* is a well established marker of epithelial cells and *MUC16*, *PAX8*, and *WT1* are considered markers of ovarian cancer [1–4]. To demonstrate the epithelial and ovarian nature of our models, we assessed these markers in the UTSW samples compared to clinical lung adenocarcinomas (LUAD), and then compared expression in the TCGA OVCA versus LUAD tumours. **A.** Normalized Illumina microarray data comparing marker gene expression in UTSW cell lines and tumours versus BCCA LUAD samples. **B.** Comparison of marker gene expression in TCGA RNA-sequencing V2 data obtained from the TCGA Data Portal (RNA-seqV2, https://tcga-data.nci.nih.gov/tcga/). In the UTSW/BCCA comparison, *EpCAM* levels were similar in LUAD and OVCA tumours, as expected given both tumour types are derived from epithelial cells. In the TCGA dataset comparison, *EpCAM* was significantly higher in LUAD although the median difference was not striking, consistent with the UTSW/BCCA comparison. In contrast, in both dataset comparisons, *MUC16*, *WT1*, and *PAX8* expression levels were significantly higher in OVCA than LUAD tumours, consistent with their expression being associated with ovarian cancer. All normalized data are presented as an expression ratio of *EpCAM*, *MUC16*, *WT1*, or *PAX8* relative to the mean of three housekeeping genes (*PSMB2*, *RAB7A*, *VCP* which were selected because they exhibited the most consistent expression levels across non-malignant samples from 429 samples, spanning 15 TCGA tissue types and the UTSW/BCCA samples) [5]. Expression ratios (ie. housekeeping gene normalization) were used to enable cross-platform and cross-tissue type comparison of expression values from our study and external datasets. Expression ratios were multiplied by a factor of 100 and plotted on a $\log_{10}$ scale for visualization. Expression levels were compared statistically using a Wilcoxin rank-sum test (U-test). P-values and median expression values are shown at the top of each figure.

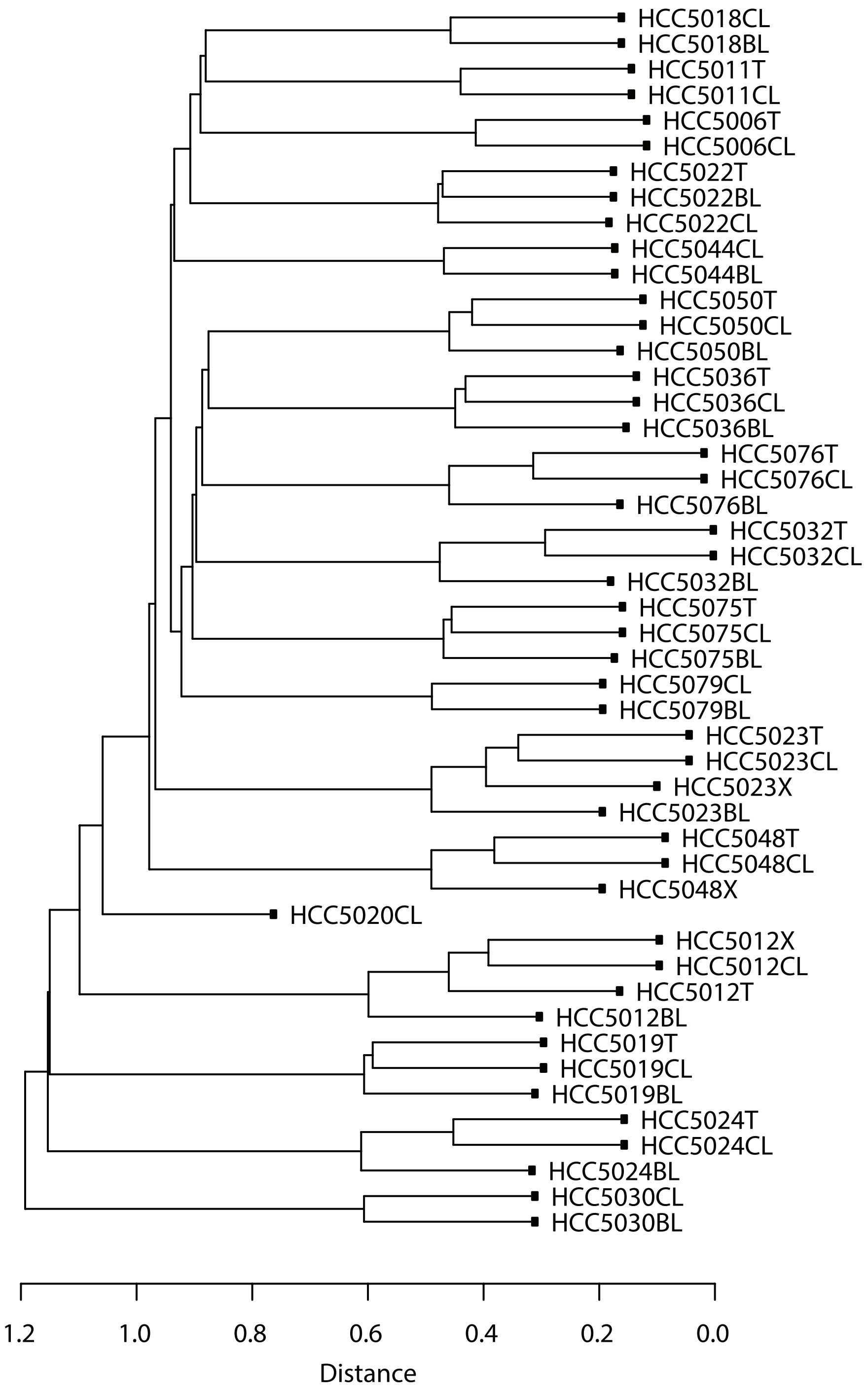

Supplementary Figure S2: Similarity in single nucleotide variant (SNV) profiles of UTSW samples. Hierarchical clustering of SNVs detected in whole exome sequencing data for 48 UTSW samples is shown. Variant calls were based on the GATK variant calling algorithm. Assessment of SNV profiles revealed as expected that individual cases clustered together. Tumor and cell line/xenograft profiles were more similar to each other than to patient-matched lymphoblast line profiles (ie. constitutional DNA) for all cases with the exception of HCC5022. We suspect this is a result of high necrotic tumor cell content and stromal cell contamination in the HCC5022 tumor ascites preparation, which was also evident by the low number of CNAs detected in HCC5022T and the poor tumor cell line CNA profile correlation. Correlation analyses of SNV data confirmed the clustering results, indicating that cell lines more closely resembled their own tumors than all other samples.

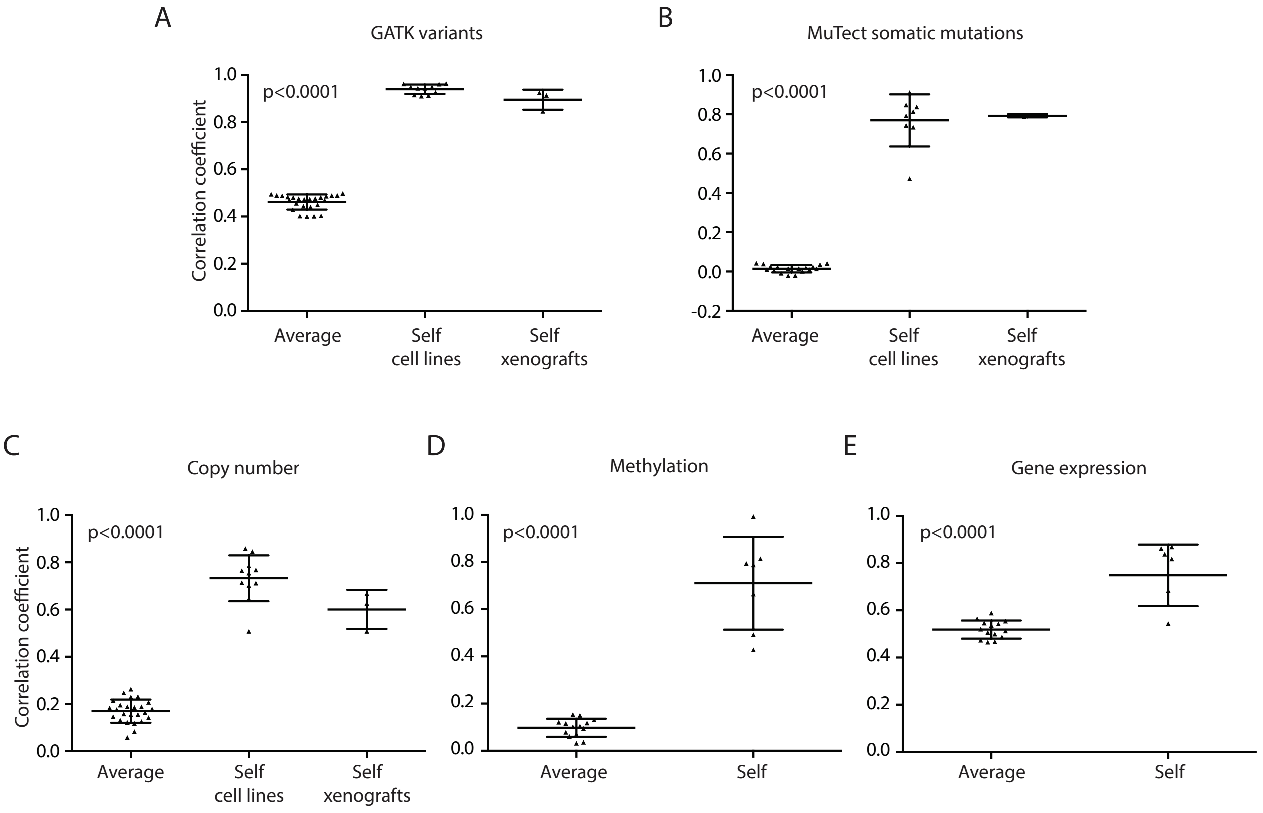

Supplementary Figure S3: Patient-matched cell line and xenograft models derived from the same tumor are more similar than they are to non-matched patient derived models. Intra- and inter-patient correlations for the various genomic dimensions characterized were performed to assess how well cell lines and xenografts mimic the genomic features of their parental tumors. For the single nucleotide variant **A., B.,** copy number **C.,** methylation **D.,** and gene expression **E.** data dimensions, the correlation between each UTSW cell line/xenograft profile and all other sample profiles (cell lines, tumors, and xenografts) was calculated. Each plot compares the genomic correlations between cell lines and xenografts and their respective tumors (Self), and their average correlation with all other samples (Average). Specifically, each dot in the "Self" plots represents the correlation between a cell line/xenograft sample and its matched tumour, while each dot in the "Average" plots represents the average correlation between an individual sample (tumour, cell line, or xenograft) and all other samples profiled for that dimension. Cell lines and xenografts were significantly more correlated to their own patient-matched tumors compared to all other samples (Student's t-test, $p<0.0001$). For MuTect variants (3B), only two xenografts were analyzed because the patient-matched non-malignant sample for HCC5048 was not exome-sequenced. For DNA methylation and gene expression, correlations were based on the 2000 most variably methylated or expressed genes. Note, methylation and expression data were not generated for xenograft samples. Four tumors (HCC5018, HCC5022, HCC5030, and HCC5032) were found to have very few CNAs (≤ 30 total) and LOH alterations detected compared to their respective cell lines (Figure 2B, Supplementary Figure 4B-4D, and Supplementary Table 7). We reasoned that the flat copy number profiles and lack of LOH calls for these tumors were attributable to low tumor cell representation relative to non-malignant stromal cells, likely due to a high proportion of necrotic tumor cells in the enriched tumor ascites. Thus, SNP array profiles (and other genomic profiles) for these tumors are highly influenced by the stable genomes of non-malignant cells present in the tumor ascites. For this reason, we excluded HCC5018, HCC5022, HCC5030, and HCC5032 cell line/tumor pairs from analyses assessing concordance between patient-matched tumor cell line genomic profiles.

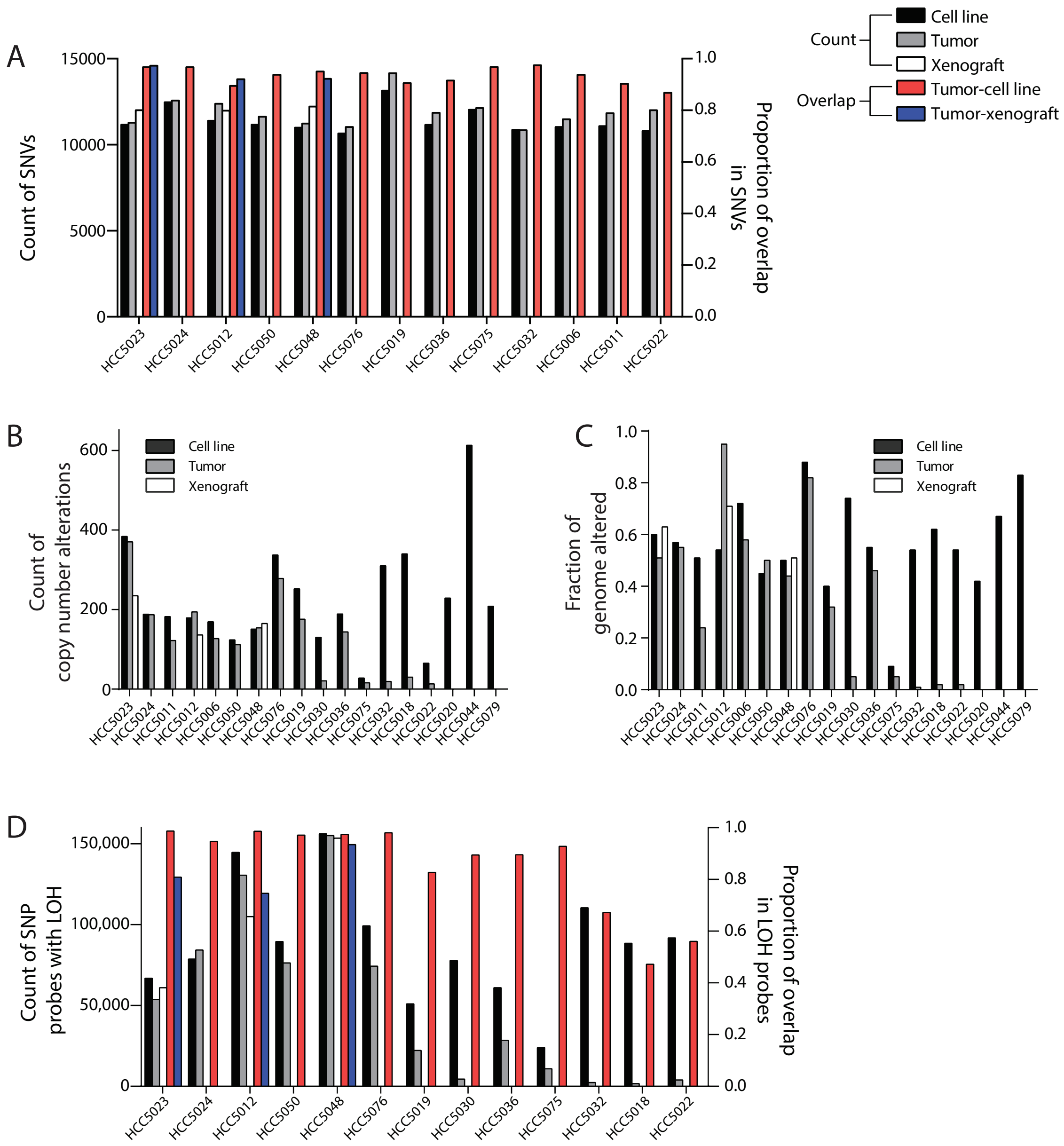

Supplementary Figure S4: Comparison of copy number, LOH, and mutational counts between matched samples. For each case, the number of single nucleotide variants (SNVs) **A.,** number of copy number alterations **B.,** fraction of genome altered **C.,** and number of SNP array probes exhibiting LOH **D.** are indicated. The proportions of overlapping SNVs and LOH events in tumors, cell lines, and xenografts are indicated in (A and D). The percentage of SNVs detected in tumors that were also detected in matched cell lines/ xenografts ranged from 87-97%, indicating the high proportion of tumor variants retained in the cell lines/xenografts generated. Likewise, the proportion of probes with LOH detected in tumors that was retained in matched cell lines and/or xenografts was very high, ranging from 75-99%. The number of samples displayed for each graph varies because not all cases included matched cell line, tumor and xenograft samples, and each data type required different combinations of samples (i.e. CNA count could be done on any somatic sample, LOH count and SNV count required cases with lymphoblast DNA for comparison). Data dimensions generated for each sample are summarized in Table 1.

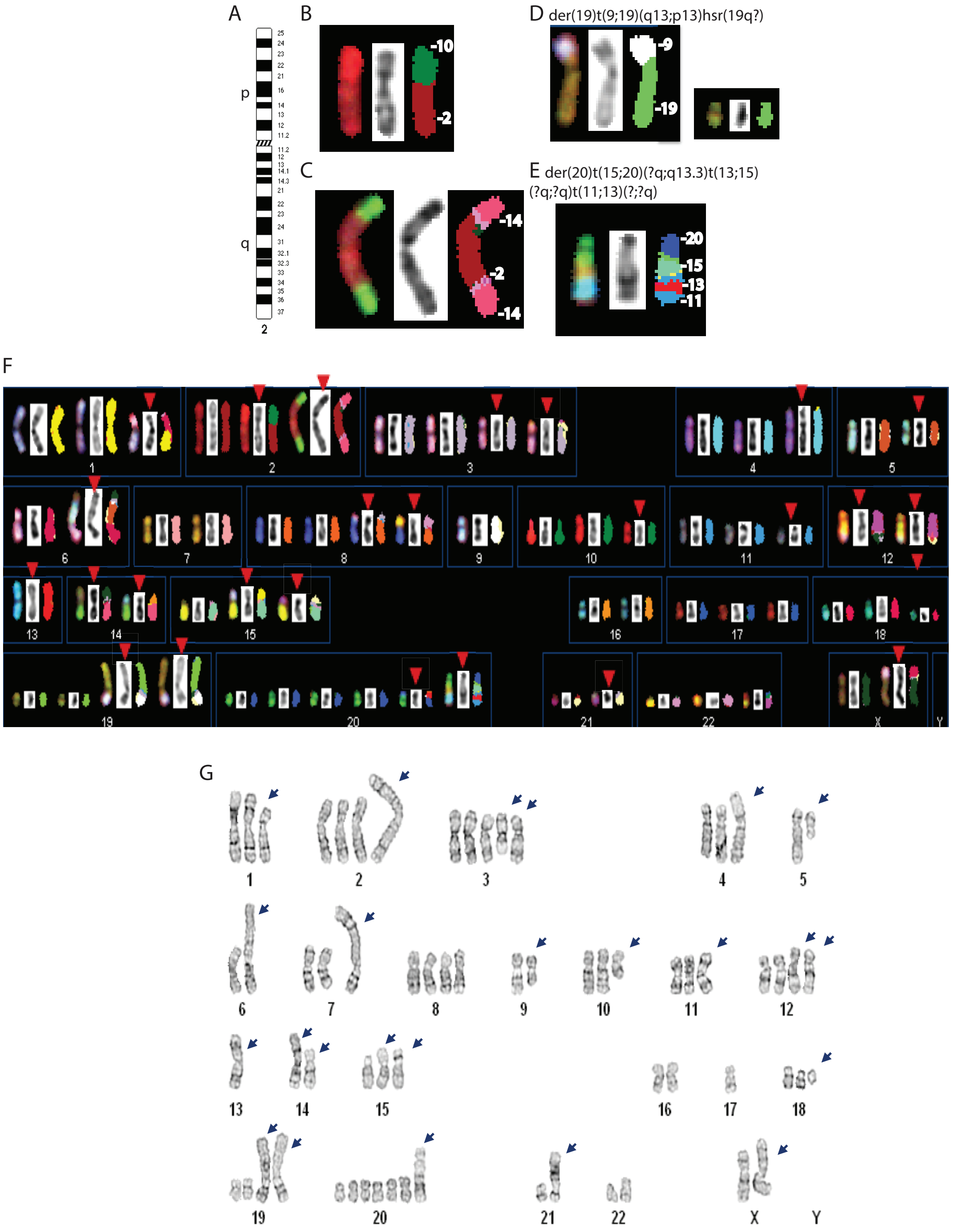

Supplementary Figure S5: Spectral karyotyping (SKY) illustrates similarity in ploidy and complex genomic rearrangements among related HCC5023 specimens. Examples of shared complex chromosomal rearrangements between the related specimens HCC5023T and HCC5023CL. **A.** Idiogram of a normal chromosome 2. **B.** derivative chromosome 2 from a translocation between chromosomes 2 and 10 with breakpoints at 2p12 and 10q21. **C.** Isoderivative chromosome 2 from a translocation between chromosomes 2 and 14 with breakpoints at 2q10 and 2q37 and at 14q11.2. **D.** Chromosome 19 on the right and derivative chromosome 19 with amplification (HSR) and t(9;19) on the left. **E.** Derivative chromosome 20 harboring a complex rearrangement involving four chromosomes. **F.** The SKY pattern for HCC5023T (left, middle and right panels for each chromosome represent respectively spectral, inverted DAPI and classified images). **G.** The GTL-banding pattern for HCC5023CL. HCC5023T shows hypo-triploid karyotype with 63 chromosomes, of which 25 were structurally abnormal. Numerical imbalances were also detected, such as copy number loss of chromosome 13 and copy number gain of chromosome 20. G-banding was performed for better identification of chromosomal breakpoints. HCC5023CL was also hypo-triploid and exhibited similar abnormalities as its partner, as indicated by the black arrows in G and red arrows in F.

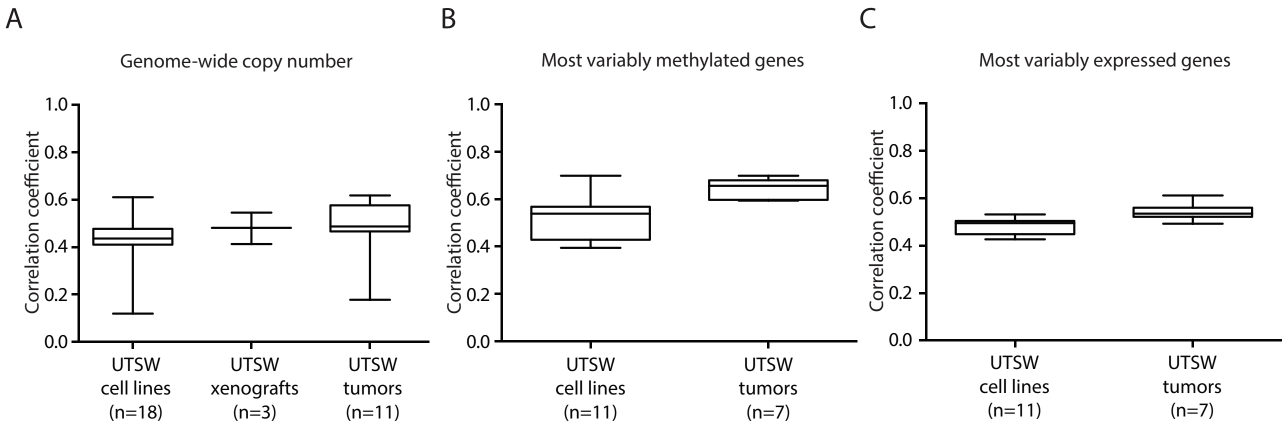

Supplementary Figure S6: UTSW cell lines resemble the TCGA HGSOC cohort across multiple genomic dimensions. Correlation coefficients for copy number **A.**, methylation **B.**, and gene expression **C.** profiles of UTSW cell lines, xenografts and tumors compared to the mean profile for each genomic dimension in the TCGA HGSOCs. Correlation coefficients were calculated using a Pearson's test. For DNA methylation and gene expression, the 2000 most variably methylated/expressed genes were considered, and tumors with low tumor relative to non-malignant content were omitted.

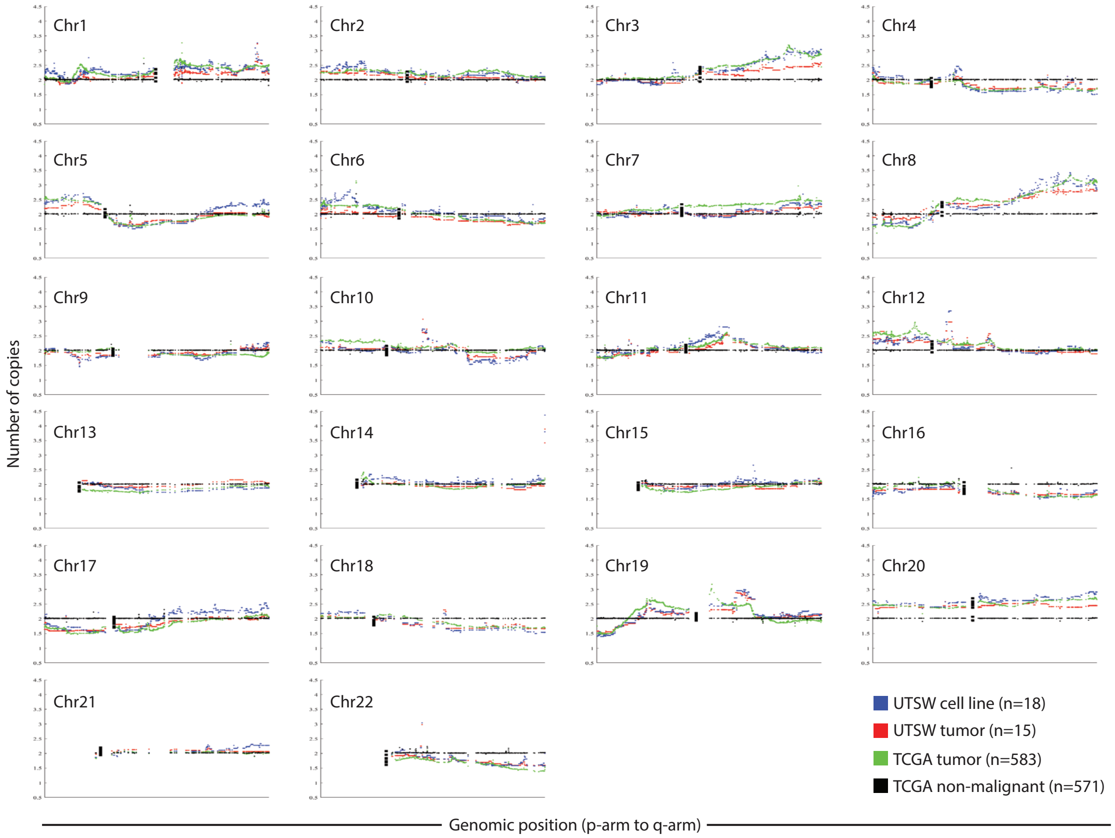

Supplementary Figure S7: Genome-wide comparison of copy number profiles for TCGA OVCA tumors and non-malignant samples, and UTSW OVCA tumors and cell lines. Copy number values (y-axis) calculated for 23,573 genes for **a.** UTSW cell lines, **b.** UTSW tumors, **c.** TCGA tumors, and **d.** TCGA non-malignant samples are plotted against genomic coordinates (x-axis) for each chromosome. Copy numbers > 2 indicate gains, while copy numbers < 2 indicate losses. A copy number of 2 is neutral (i.e. no copy number change). The overlay in samples demonstrates the high concordance in copy number changes throughout the genome for TCGA HGSOC tumors and the UTSW samples, relative to non-malignant (copy neutral) tissue-matched controls.

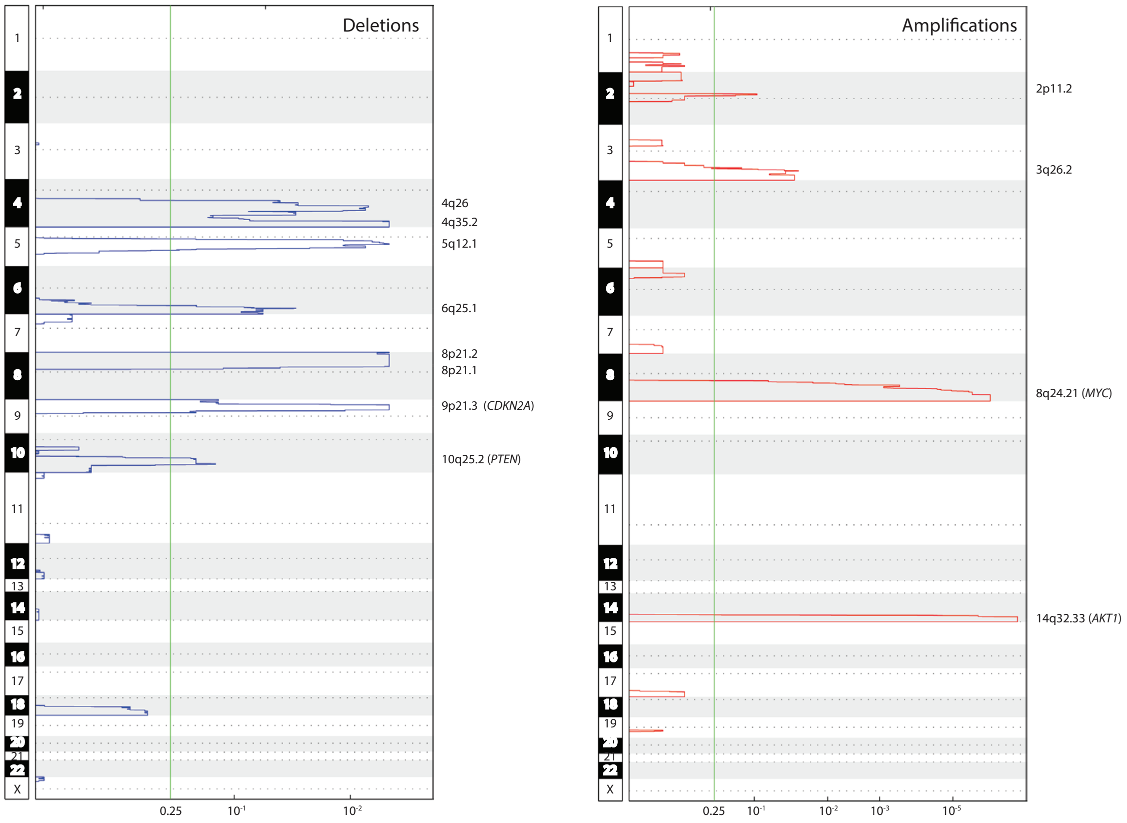

Supplementary Figure S8: Significant regions of CNA identified by GISTIC. The GISTIC algorithm was performed to identify recurrent, high magnitude copy number changes in the 18 UTSW cell lines. Significant regions of deletion and amplification are illustrated, with peak regions indicated. A summary of the regions identified is provided in Supplementary Table 9 and the genes affected are presented in Supplementary Table 10. Genomic coordinates supplied are those for the hg18 genome build to facilitate comparison to the TCGA OVCA data.

Supplementary Table S1: Cell line culture characteristics

See Supplementary File 1

Supplementary Table S2: STR profiles of UTSW cases

See Supplementary File 2

Supplementary Table S3: CA125 levels in patient serum and derived cancer cell lines

See Supplementary File 3

Supplementary Table S4: Variants detected by exome-sequencing

See Supplementary File 4

Supplementary Table S5: MuTect variants

See Supplementary File 5

Supplementary Table S6: Exome-sequencing summary statistics

See Supplementary File 6

Supplementary Table S7: Summary of copy number alterations detected

See Supplementary File 7

Supplementary Table S8: SKY-derived chromosomal ploidy and frequency of clonal structural and numerical abnormalities

See Supplementary File 8

Supplementary Table S9: GISTIC copy number regions

See Supplementary File 9

Supplementary Table S10: Genes in GISTIC regions

See Supplementary File 10

Supplementary Table S11: Cell line response to cisplatin and paclitaxel

See Supplementary File 11
